# Different Performance of Intravertebral Vacuum Clefts in Kümmell's Disease and Relevant Treatment Strategies

**DOI:** 10.1111/os.12609

**Published:** 2020-02-19

**Authors:** Wei Wang, Qian Liu, Wei‐jun Liu, Qing‐bo Li, Lei Cai, Zheng‐kun Wang

**Affiliations:** ^1^ Department of Orthopaedics, Wuhan Fourth Hospital, Puai Hospital, Tongji Medical College Huazhong University of Science and Technology Wuhan Hubei China; ^2^ Department of Plastic and Cosmetic, Wuhan Tongji Hospital, Tongji Medical College Huazhong University of Science and Technology Wuhan Hubei China

**Keywords:** Intravertebral vacuum cleft (IVC), Kümmell's disease, Long‐segment fixation (LSF), Percutaneous kyphoplasty (PKP), Posterior short‐segment fixation (SSF)

## Abstract

**Objectives:**

This study aimed to present the different pattern of intravertebral vacuum cleft (IVC) related to high risk of cement complications in minimally invasive treatments for Kümmell's disease (KD) and relevant treatment strategies.

**Methods:**

A retrospective study from January 2016 to January 2018 was conducted at Wuhan Fourth Hospital and comprised 35 patients with Kümmell's disease. There were seven males and 28 females, and the mean age of the patients was 70.4 years. The patterns of IVC in KD were analyzed. These patients were divided into three groups based on the treatment method used. The treatment methods included long‐segment fixation (LSF), posterior short‐segment fixation (SSF), and percutaneous kyphoplasty (PKP). We retrospectively reviewed outcomes, including the Oswestry Disability Index (ODI), visual analog scale (VAS) score, anterior height of affected vertebrae, kyphotic Cobb angle, and complications.

**Results:**

All patients were followed up for 12–38 months. According to their radiographic appearance we could observe two main patterns of clefts. Pattern I, clefts that were found to be near to the endplate and connected with intervertebral space, the endplate was incomplete. Pattern II, IVC traversed to anterior edge of the vertebral body affected. Both were related to high risk of cement complications in minimal invasive treatments for KD. Good results have been achieved in LSF and SSF groups, the VAS, ODI, anterior height of affected vertebrae and kyphotic Cobb angle showed statistically significant differences between pre‐ and post‐operation and between pre‐ and final follow‐up (*P* < 0.05). In PKP group, although the VSA and ODI showed statistically significant differences between pre‐ and post‐operation and between pre‐ and final follow‐up (*P* < 0.05), we could observe that the VSA and ODI rebounded a little at the final follow‐up. Cement leakage into intervertebral space occurred in four (44.45%) patients of PKP group.

**Conclusions:**

PKP should be chosen carefully if the IVC of the patient presents to be pattern I or II. LSF and SSF are safe and effective, and can achieve satisfactory correction of kyphosis and vertebral height, with pain relief and improvement in patient's daily life, with few complications.

## Introduction

Following the accelerated speed of population aging, osteoporotic vertebral compression fracture (OVCF) has become increasingly common. Among which the incidence of nonunion is approximately 13.5% and the incidence of intravertebral vacuum cleft (IVC) sign is about 7%–13%^1^. Kümmell's disease (KD), or avascular necrosis of a vertebral body, presents as vertebral osteonecrosis with compression deformity, IVC, exaggerated kyphosis and intravertebral instability weeks to months after a minor trauma^2^.

In 1895, German surgeon Hermann Kümmell first reported six patients with delayed posttraumatic vertebral collapse after minor trauma, which was referred to as KD^3^. While initially KD was thought to be exceedingly rare, its prevalence is increasing with our aging and often osteoporotic population, and our recognition of this disease.

Up to now, there are several studies on classification and staging of KD, all according to the disease's progression. Dr Steel divided KD into five stages according to the development of symptoms. Stage 1 is the initial insult with no abnormality on the X‐ray. Stage 2 is the post‐traumatic period of minor back pain. Stage 3 represents the latent interval of relative well‐being; patients in this stage usually experience asymptomatic period lasting from months to years. Stage 4 means the recrudescent stage; patients may feel progressive pain at the fracture position. Stage 5 is the terminal stage; patients may suffer permanent kyphosis or compression of roots and spinal cord in this stage^4^. Ito *et al*.^5^ postulated that the pathogenesis of progression to delayed vertebral collapse could be divided into three stages. Stage 1: intravertebral cleft appearance (3 weeks after). Stage 2: morphological intravertebral cleft changes and intravertebral instability of the affected vertebra. Stage 3: complete vertebral collapse. According to the clinical symptoms, radiographs, and the MRI, Li *et al*.^6^ divided the KD into three stages. Stage I: minor compression with or without back pain. Stage II: vertebral body height loss >20%, back pain with or without radiculopathy. Stage III: posterior cortex breakage leading to cord compression with or without cord injury.

No standard or single effective treatment for Kümmell disease exists. In general, conservative treatments have usually been less effective, and carry a risk for delayed neurological deficits^7^, ^8^. Most surgeons have suggested that Kümmell disease should be treated by operative intervention. Traditionally, early stage of the disease can be treated by minimally invasive treatments, including vertebroplasty (PVP) or kyphoplasty (PKP). Several studies showed that PKP and PVP could achieve good pain relief, vertebral body height restoration, and kyphosis deformity correction^9^, ^10^. Restoring physical stability and eliminating motion at the intravertebral vacuum cleft by cementing is the most important pain relief mechanism in Kümmell's disease^11^, ^12^. Fixation surgery and body reconstruction should be considered in treating stage III KD^13^, ^14^, ^15^. However, with the increasing cognition of the disease and wide application of minimally invasive treatments, more and more complications of minimally invasive treatments, including cement extravasation, fractured transverse processes or ribs, infection, hematomas, and subsequent fracture of adjacent vertebral body, have been reported^4^, ^16^, ^17^. On the other hand, some surgeons thought PKP is an effective, minimally invasive procedure for the treatment of chronic osteoporotic stage III Kümmell's disease even with severe spinal stenosis^18^.

By referring to various articles, combined with our experience on treatment of the disease, we recognized that the cement leakage, extravasation or dislodge of PVP/PKP has relation with the pattern of IVC in the vertebral body, rather than the stage of the disease. Therefore, this retrospective study aimed to provide surgeons with: (i) the different patterns of IVC related to the high risk of cement complications in minimally invasive treatments for KD; (ii) useful and reasonable treatments to reduce the incidence of cement complications; and (iii) classification and treatment strategies of KD based on the different patterns of IVC rather than the stage of the disease.

The patterns of IVC in KD were analyzed. Patients were divided into three groups based on the treatment method used. The treatment methods included long‐segment fixation (LSF), posterior short‐segment fixation (SSF), and percutaneous kyphoplasty (PKP). We retrospectively reviewed outcomes, including the Oswestry Disability Index (ODI), visual analog scale (VAS) score, anterior height of affected vertebrae, kyphotic Cobb angle, and postoperative complications.

## Patients and Methods

### *Patient Demographics*

Each patient provided informed consent for participation in the study. This retrospective study was conducted in accordance with the Declaration of Helsinki (Ethical Principles for Medical Research Involving Human Subjects) and was approved by the Ethics Committee of the Wuhan Fourth Hospital, Huazhong University of Science and Technology.

From January 2016 to January 2018, the inclusion criteria were as follows: (i) had a history of minor trauma or had no memorized accident; (ii) back pain weeks to months after the initial minor traumatic injury; (iii) IVC signs on simple lateral radiographs and computed tomography (CT) or evidence of osteonecrosis with a fluid sign on magnetic resonance imaging (MRI); and (iv) conservative treatment failed to relieve the pain. Exclusion criteria: (i) congenital lumbar scoliosis, spinal stenosis, or other deformities; (ii) pathologic vertebral compression fractures from spinal metastatic cancer, osteolytic bone tumors, and hemangioma; and (iii) a history of previous vertebroplasty or spinal surgery. Finally, 35 KD patients (seven males and 28 females) were included.

All the pre‐ and postoperative radiographs of these patients at our medical center were analyzed retrospectively.

All patients underwent plain film, dynamic x‐ray, computed tomography (CT) scans, and magnetic resonance imaging (MRI).

The mean age of the patients was 70.4 years, ranging from 61 to 84 years. The patients had different degrees of osteoporosis, and the lumbar spine bone mineral density T score (BMD) ranged from −2.9 to −4.4, with a mean of −3.5 ± 0.8. Eight participants had no memorized accident, while the other 27 presented minor trauma, including sprain (13 cases), tumble (seven cases), fall (five cases) and bruise (two cases). There were 38 vertebral bodies affected, with three patients being double‐level vertebral affected, and 91.43% were single‐level vertebral‐body affected cases. All patients presented intractable back pain. The timing of back pain ranged from 1 month to 9 months, with an average of 4.3 months. Changes in position induced or aggravated the pain, which could not be relieved by bed rest or regular medical treatment with drugs (such as Celecoxib) for 3 months. All patients were followed up for 12–38 months.

### *Patterns of IVC and Classification*

**Figure 1:**
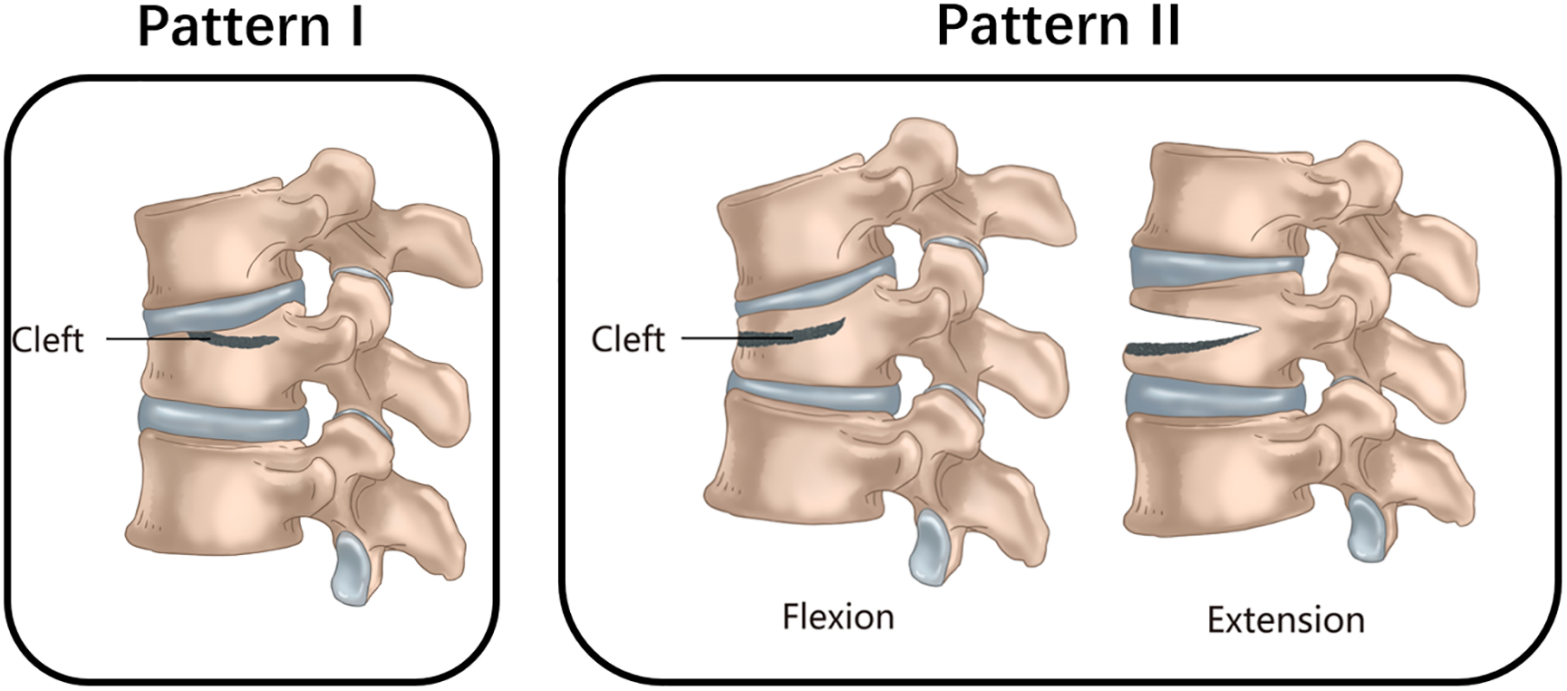
This illustration shows the two main patterns of clefts in KD. Pattern I, clefts that were found to be near to the endplate and connected with intervertebral space (arrow), the endplate was incomplete. Pattern II, IVC traversed to anterior edge of the vertebral body affected. Most cases of this pattern are very dynamic instable, for the nonunion run through the anterior column even anterior and middle columns let the remaining rear structure of the vertebrae may act as a hinge when the spine flexes forward and extends backward.

According to their radiographic appearance, we could observe two main patterns of clefts: Pattern I, clefts that were found to be near to the endplate and connected with intervertebral space, the endplate was incomplete; and pattern II, IVC traversed to anterior edge of the vertebral body affected. These patterns are shown in Fig. 1. According to the clinical symptoms, these patients can be divided into two groups. Patients are considered type A if they have any of the following conditions: neurological deficits requiring decompression, kyphosis deformity requiring correction (Cobb angle greater than 30°), or dynamic instability (the difference of Cobb angle during dynamic radiography greater than 10°) requiring fixation operation. If the patients only suffered from intractable back pain without any of these conditions, they were considered type B.

### *Treatment Strategies*

The general principle of treatment for KD was to enhance and support anterior column, and to fix and stabilize posterior column. The treatment methods included open surgery and minimally invasive treatment; the former included posterior long‐segment fixation (LSF) and posterior short‐segment fixation (SSF) based on the different condition, while the latter referred to PKP according to our therapeutic experience in this study.

Basically, LSF was performed in all type A patients, while SSF and PKP were optional in type B patients. Type B patients decided themselves which treatment to choose after being fully informed of the advantages, disadvantages and complications of these two treatment methods, respectively.

Then the 35 patients were divided into three groups (LSF, SSF, and PKP) based on the surgical method used.

## Surgical Procedures

### *Posterior LSF*

**Figure 2:**
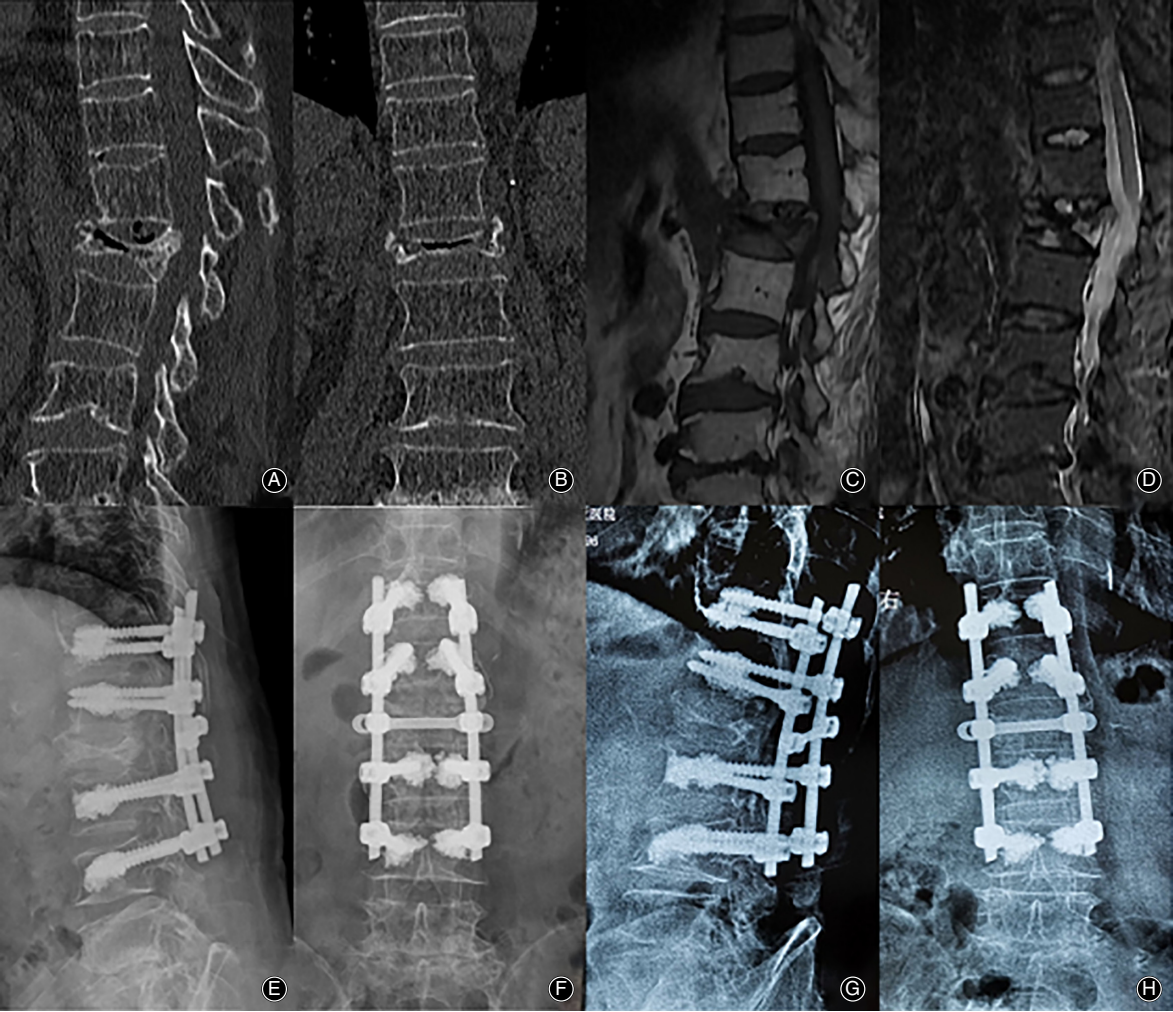
A 67‐year‐old‐female patient who underwent LSF for L_2_ Kummell disease. (A‐B) Preoperative CT scan showing a L_2_ burst fracture with IVC, and MRI showing low signal of the necrotic area and spinal cord compression on the T1(C) and T2‐weighted (D) images. Postoperative (E and F) and final follow‐up (G and H) X‐rays revealing good internal fixation position and satisfactory radiographic results.

The patients in the LSF group received general anesthesia and were placed in a prone position. A posterior midline approach was used to expose the spinous processes, lamina, and facet joint; eight or 12 pedicle screws were inserted bilaterally into two or three levels above and below the affected vertebra. Decompressive laminectomy of the diseased vertebra was applicable if patients had neurologic deficits; pedicle subtraction osteotomy (PSO) or Ponte osteotomy was applicable to correct the kyphotic deformities; cement‐augmented pedicle screws were used to address the high risk of internal fixation failure according to severe osteoporosis. Calcium sulfate with BMP (Rebone biomaterials CO., Shanghai, China) was injected slowly into the diseased vertebra by cannula and trocar systems to fill the IVC to enhance the anterior column, and this process was monitored under fluoroscopic control in the lateral plane. After that, pedicle screws were fixed with two rods, which were used to achieve further recovery of the vertebral height. Long‐segment fixation was used to restore sagittal balance and stabilize the spinal column. Autogenous bone grafts from the decompression laminectomy were used to facilitate posterolateral fusion. Crosslinks were used in all patients. The incision was rinsed and hemostasis achieved, then was sutured hierarchically (Fig. 2).

### *Posterior SSF*

**Figure 3:**
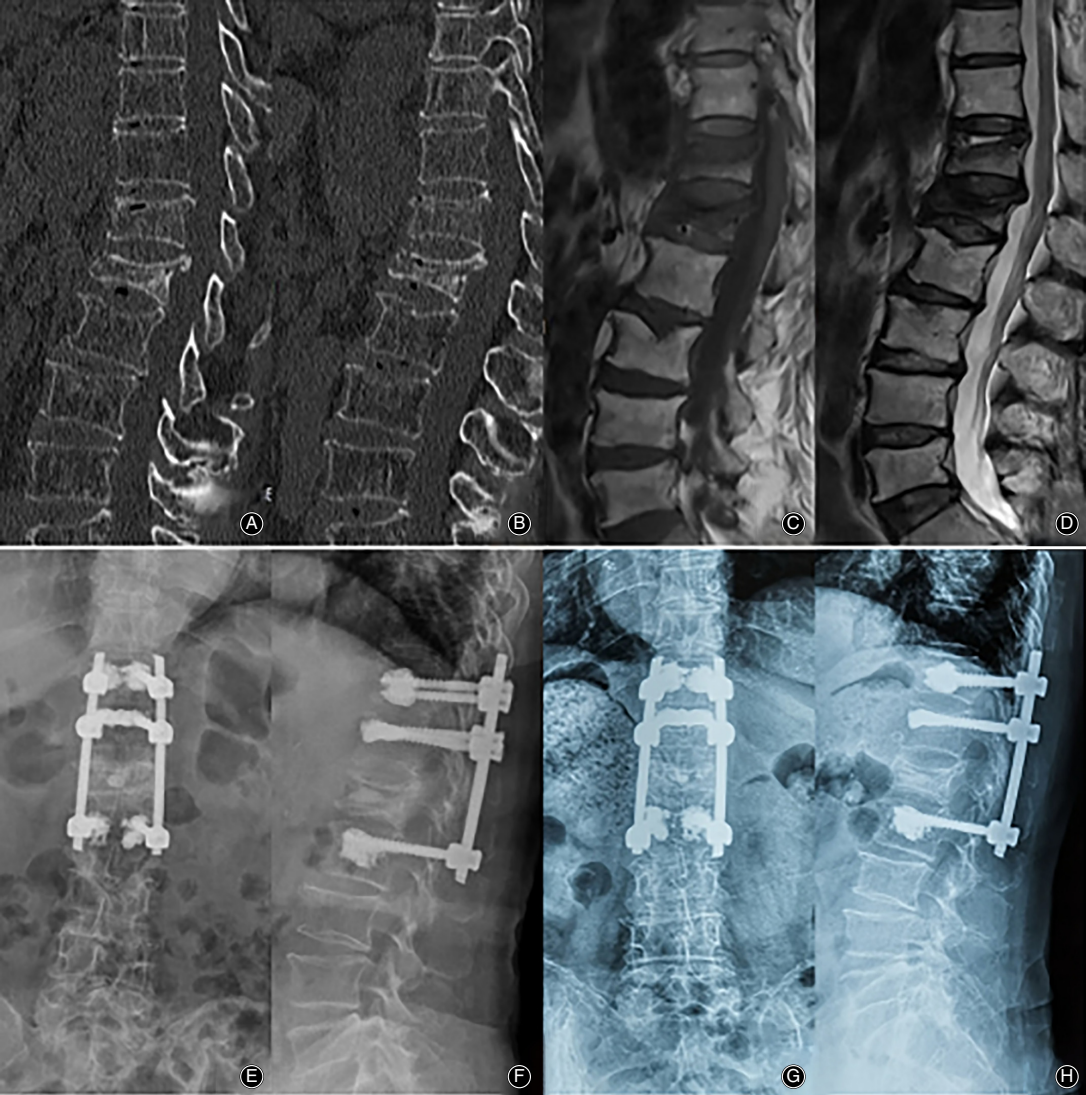
A 76‐year‐old female patient who underwent SSF for T_12_ and L_1_ Kummell disease. (A‐B) Preoperative CT scan showing T_12_ and L_1_ fracture with IVC, and MRI showing IVC on the T1(C) and T2‐weighted (D) images. Postoperative (E and F) and final follow‐up (G and H) X‐rays revealing good internal fixation position and satisfactory radiographic results.

The patients in the SSF group received general anesthesia and were placed in a prone position. Standard posterior midline approach was performed, as described in LSF. Four pedicle screws were inserted bilaterally into affected vertebra and one level above, or one level above and one level below the affected vertebra. Cement‐augmented pedicle screws were used if the patient suffered severe osteoporosis. Calcium sulfate with BMP (Rebone biomaterials CO., Shanghai, China) was injected slowly into the affected vertebra like the procedure described in LSF. After that, pedicle screws were fixed with two rods, which were used to achieve further recovery of the vertebral height. Decompressive laminectomy of the diseased vertebra was performed for patients with neurological deficits. Posterolateral fusion was performed by the application of allograft bone. The incision was dealt with by the same procedure described in LSF (Fig. 3).

### *PKP*

**Figure 4:**
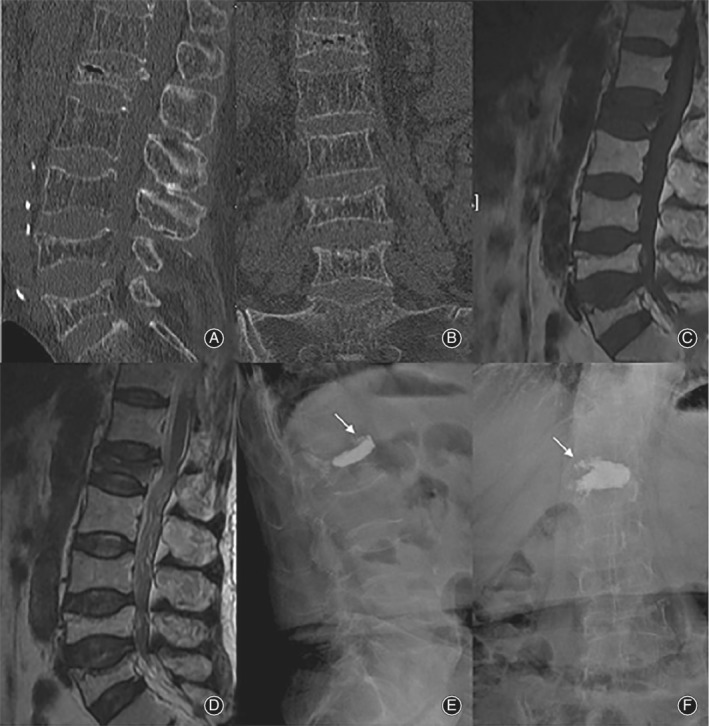
A 65‐year‐old‐female patient who underwent PKP for L_1_ Kummell disease. (A and B) Preoperative CT scan showing L_1_ fracture with IVC, and MRI showing IVC on the T1(C) and T2‐weighted (D) images. Postoperative X‐rays (E and F) revealing radiographic results, which shows cement leakage at T_12_ L_1_ disk space (arrow).

The patients in the PKP group received local anesthesia (1% lidocaine, 4 mL) and were placed in a prone position. A transpedicular approach was performed with trocar and cannula systems under biplanar fluoroscopic guidance. The expandable balloon was placed into position. The ideal position of the top of the needle was laterally positioned in the first quarter of the interior of the affected vertebrae and the channel went through IVC. The balloons were expanded up slowly to the expected effect of vertebral reset and to create a cavity for the injected cement. Polymethyl methacrylate (PMMA) cement was injected into the cavity within the vertebral body when it became doughy and could stand at the tip of the bone cement inserter. The injection process was monitored continuously under fluoroscopic control in the lateral plane. The procedure was stopped immediately if high resistance was encountered or if PMMA neared the posterior wall of the vertebral body. The amount of cement was generally 4–8 mL. We continued to observe the distribution of the bone cement in the vertebrae. The curing time of bone cement was 15 min, and after waiting sufficient time and confirming bone cement curing, we turned and pulled out the pipe. The patients were kept at bed rest for 1 day after the procedure (Fig. 4).

### *Clinical Assessment*

The impact on the patient's daily life was assessed using the Oswestry Disability Index (ODI) questionnaire. The visual analogue scale (VAS score 0–10; 0 no pain; 10 the worst imagined) system was employed to evaluate back pain control. All patients were evaluated preoperatively, at 7 days postoperatively, and at the final follow‐up.

### *Oswestry Disability Index*

The ODI is one of the principal condition‐specific outcome measures used in the management of spinal disorders. For each section of six statements, the total score is five (the first statement is marked with the score “zero” and the last statement is marked as “five”). If more than one box is marked in each section, the highest score is taken. If all 10 sections are completed, the score is calculated as follows: total scored/50 (total possible score) × 100%. If one section is missed (or not applicable), the score is calculated as: total scored/45 (total possible score) × 100%. Therefore, the final score may be summarized as: [total score/(5 × number of questions answered)] × 100%. Rounding the percentage to a whole number is suggested for convenience. We defined that: 0%–20% means mild; 21%–40% means moderate; 41%–60% means severe; 61%–80% means very severe; and 80%–100% means patients with very exaggerated symptoms.

### *Visual Analogue Scale*

The VAS score system is used in the social and behavioral sciences to measure low back pain and leg pain. The VAS pain scoring standard (scores from 0 to 10) was as follows: 0 means painless; 1–3 means mild pain that the patient could endure; 4–6 means the patient was in pain that could be endured and was able to sleep; and 7–10 means the patient had intense pain and was unable to tolerate the pain.

### *Radiologic Assessment*

All patients underwent plain film, dynamic x‐ray, CT scans, and MRI. The anterior height of the affected vertebrae along with the kyphotic angle was measured on a lateral x‐ray. Cobb's methods were used to measure the kyphotic angle of the affected vertebrae. Dynamic X‐ray was used to access spine dynamic instability, which was defined as the difference of Cobb angle during dynamic radiography greater than 10°. All of the foregoing data were evaluated preoperatively, at 7 days postoperatively, and at the final follow‐up. Complications were recorded as well.

### *Statistical Analysis*

All analyses were performed using SPSS for Windows, version 19.0 (IBM, Armonk, New York, USA). All measurement data were expressed as mean and standard deviation (SD). Preoperative and postoperative measurement data, including VAS, ODI score, anterior height of fractured vertebrae, and kyphotic Cobb angle were compared using paired *t*‐test or one‐way ANOVA. Count data, including sex and damaged segment, were compared using chi‐square test or Fisher exact test. A *P* value <0.05 was considered to indicate a statistically significant difference.

## Results

### *Demographic Data*

There were 20 pattern I and 15 pattern II patients respectively. No statistically significant difference was observed in the general data (age, gender, medical history, damaged segment, bone mineral density) between these two IVC patterns (Table 1).

**Table 1:** Patient data in IVC patterns

Variable	Pattern I	Pattern II	*P* value
Number of patients	20	15	
Age(years), mean ± SD	70.2 ± 7.8	69.5 ± 6.8	0.782
Gender, female/male, number	16/4	12/3	0.854
Medical history(months), mean ± SD	4.2 ± 1.5	3.9 ± 1.9	0.597
BMD T score, mean ± SD	−3.9 ± 0.6	−3.6 ± 0.7	0.643
Damaged segment, TL/NTL, number	19/2	16/2	0.739

Statistical significance is set at *P* < 0.05

BMD, bone mineral density; LSF, long‐segment fixation; NTL, affected vertebra located not in thoracolumbar junction; SD, standard deviation; SSF, short‐segment fixation; TL, affected vertebra located in thoracolumbar junction

Twenty‐six patients received open surgery, among which 14 were SSF and 12 were LSF. PKP was performed in nine type B patients. These general data also had no statistically significant difference among the three different treatment groups (Table 2).

**Table 2:** Patient data in treatment groups

Variable	LSF group	SSF group	PKP group	*P* value
Number of patients	12	14	9	
Age (years), mean ± SD	71.2 ± 5.9	70.3 ± 7.8	69.2 ± 8.5	0.895
Gender, female/male, number	10/2	11/3	7/2	0.533
Medical history (months), mean ± SD	4.2 ± 1.9	4.1 ± 1.7	3.9 ± 1.7	0.409
BMD T score, mean ± SD	−4.0 ± 0.7	−3.5 ± 0.8	−3.4 ± 0.6	0.538
Damaged segment, TL/NTL, number	12/1	14/2	8/1	0.627

Statistical significance is set at *P* < 0.05

BMD, bone mineral density; LSF, long‐segment fixation; NTL, affected vertebra located not in thoracolumbar junction; SD, standard deviation; SSF, short‐segment fixation; TL, affected vertebra located in thoracolumbar junction

### *Clinical and Radiological Findings*

Good results have been achieved in LSF and SSF groups, the VAS, ODI, anterior height of affected vertebrae and kyphotic Cobb angle showed statistically significant differences between pre‐ and post‐operation and between pre‐ and final follow‐up (*P* < 0.05; Tables 3 and 4), whereas the differences between postoperative and final follow‐up values were not statistically significant (*P* > 0.05; Tables 3 and 4).

**Table 3:** Evaluation index in LSF group

Time	VAS score	ODI (%)	Anterior vertebral height (mm)	Kyphotic Cobb angle (°)
Pre‐operation	9.3 ± 1.1	87.9 ± 8.3	8.5 ± 2.1	40.8 ± 4.5
Post‐operation	2.4 ± 1.0 *	36.5 ± 5.3 *	27.4 ± 3.3 *	9.3 ± 2.1 *
Final Follow‐up	2.3 ± 0.9 * †	37.2 ± 4.9 * †	27.2 ± 2.6 * †	9.1 ± 1.8 * †

Statistical significance is set at *P* < 0.05

ODI, Oswestry Disability Index; VAS, visual analog scale

* *P* < 0.05 *vs*. pre‐operation values

† *P* > 0.05 *vs*. post‐operation values

**Table 4:** Evaluation index in SSF group

Time	VAS score	ODI (%)	Anterior vertebral height (mm)	Kyphotic Cobb angle (°)
Pre‐operation	8.9 ± 1.2	84.5 ± 5.9	15.4 ± 3.6	19.3 ± 2.8
Post‐operation	2.1 ± 0.8 *	32.9 ± 3.7 *	23.3 ± 2.6 *	11.6 ± 1.5 *
Final Follow‐up	2.2 ± 0.7 * †	31.1 ± 3.4 * †	23.1 ± 1.7 * †	11.3 ± 0.9 * †

Statistical significance is set at *P* < 0.05

ODI, Oswestry Disability Index; VAS, visual analog scale

* *P* < 0.05 *vs*. pre‐operation values

† *P* > 0.05 *vs*. post‐operation values

In PKP group, the VSA and ODI showed statistically significant differences between pre‐ and post‐operation and between pre‐ and final follow‐up (*P* < 0.05; Table 5), whereas the differences between postoperative and final follow‐up values were also statistically significant (*P* < 0.05; Table 5), as cement leakage and recurrent lower back pain occurred during follow‐up. We could observe that the VSA and ODI rebounded a little at the final follow‐up. The differences of anterior vertebral heights and kyphotic Cobb angle at each time point were not statistically significant (*P* > 0.05; Table 5).

**Table 5:** Evaluation index in PKP group

Time	VAS score	ODI (%)	Anterior vertebral height (mm)	Kyphotic Cobb angle (°)
Pre‐operation	9.1 ± 0.7	77.5 ± 7.3	20.7 ± 2.3	8.9 ± 1.2
Post‐operation	2.1 ± 0.9 *	31.5 ± 4.4 *	21.1 ± 2.1 §	8.3 ± 1.1 §
Final Follow‐up	3.9 ± 0.5 * †	47.1 ± 3.9 * †	21.2 ± 1.9 § †	8.1 ± 0.7 § ‡

Statistical significance is set at *P* < 0.05

ODI, Oswestry Disability Index; VAS, visual analog scale

* *P* < 0.05 *vs*. pre‐operation values

† *P* < 0.05 *vs*. post‐operation values

‡ *P* > 0.05 *vs*. post‐operation values

§ *P* > 0.05 *vs*. pre‐operation values

### *Complications*

No serious intraoperative or postoperative complications were observed in all these patients. One patient in the LSF group and one patient in the SSF group had delayed wound healing, one patient in the LSF had a urinary tract infection, and they were all cured by conservative treatment. Cement leakage into intervertebral space occurred in four (44.45%) patients of PKP group; among them, three patients had recurrent lower back pain during the 3–9 months after the procedure. Their pain were all tolerable and could be relieved by bed rest or regular medical treatment with drugs, VAS score was 3 to 5. No obvious bone cement displacement occurred during the follow‐up. There was no loosening, displacement, or fracture of internal fixation at the final follow‐up. No neurologic deterioration was seen after the surgery.

## Discussion

KD is defined as delayed post‐traumatic vertebral collapse after a minor spinal trauma and often occurs in patients with osteoporosis, advanced age, or those undergoing long‐term steroid therapy. Single vertebral body is involved commonly and the most common fractured and necrotic vertebral body is located in the thoracolumbar junction^12^, ^18^, ^19^. In this study, the mean age of the patients was 70.4 years, the average BMD was −3.5 ± 0.8, 91.43% (32 in 35) were single‐level vertebral body affected cases, and 90% affected vertebral body was located in the thoracolumbar junction. These findings were all consistent with these previous studies^12^, ^18^, ^19^ and were in accordance with features of KD.

### *Challenging for Management of KD*

While initially KD was thought to be exceedingly rare, its prevalence is increasing with our aging and often osteoporotic population, and our recognition of this disease. Up to now, there is not yet a consensus reached on classification and corresponding treatment options for KD. Traditionally, KD could be divided into three stages according to clinical symptoms, radiographs, MRI and the progression of the disease^6^. Minimally invasive treatments are applicable for the early stage of the disease, while late‐stage KD may present with cortical breakage and cord compression, which are contraindicative for cement augmentation^20^. Open surgery, including fixation surgery and body reconstruction, should be considered in treating the late stage of the disease.

However, this management of KD remains controversial and there are no strict guidelines of treatment strategies for work‐up or treatment. Some authors have reported that PVP/PKP was found to be a minimally invasive and effective procedure that provides pain relief and stabilization of spinal instability associated with KD, even in the late stage of the disease with severe spinal stenosis^18^. The IVC would be filled with bone cement and stabilized to achieve therapeutic purpose because unstable fractures and motion within the IVC produces severe and persistent pain^21^. On the other hand, some authors insisted that bone cement augmentation for treating KD is associated with recurrent vertebral collapse as well as dislodged or fragmented bone cement^22^. In the meantime, more and more complications of PVP/PKP are reported even in the early stage application. This literature seemed to prove that cement complications during minimally invasive treatments have little relationship with the stage of KD.

### *Patterns of IVC and Treatment Strategies*

By referring to various research, combined with our experience with the treatment of the disease, we recognized that what is even more concerning is the pattern of IVC in the vertebral body, rather than the stage of the disease. In this study, we observed and summarized the different performance of IVC from 35 KD patients, and noted two main patterns of clefts that existed.

**Figure 5:**
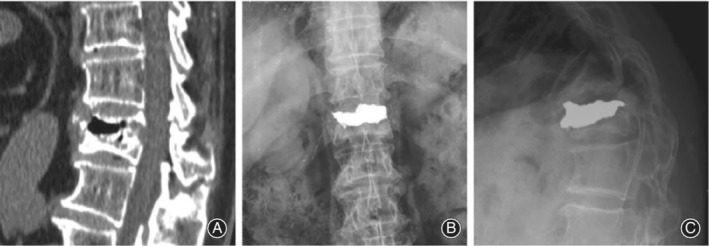
A typical patient with pattern I IVC from Wu *et al*.^16^. (A) Preoperative CT show the IVC near to the endplate.(B) cement leak into intervertebral disk. (C) adjacent vertebral body fracture 3 months after the first surgery.

Pattern I, clefts that were found to be near to the endplate and be connected with intervertebral space, the endplate was incomplete. In this pattern, cement is difficult to disperse throughout the cleft, but easily leaks into the intervertebral space through the cleft for the integrity of the endplates were destructed, which may cause discogenic back pain later on. As showed in Fig. 4, cement leakage was found after PKP. Cement leakage into intervertebral space occurred in four patients of PKP group; among them, three patients had recurrent lower back pain 3–9 months after the procedure. Armingeat *et al*. noted that the IVC and intradiscal vacuum cleft often co‐exist, and a communication channel could be observed between them^23^. Cement was readily leaking into intervertebral disk via the channel. Recently, intradiscal cement leak has been shown to increase the risk of subsequent new fractures of adjacent vertebral bodies^24^, ^25^, ^26^. Wu *et al*.^27^ reported a typical case demonstrating the coexistence of IVC and intradiscal vacuum cleft, which showed cement leak into the intervertebral disk, and an adjacent vertebral body fracture at 3 months after the first operation (Fig. 5).

**Figure 6:**
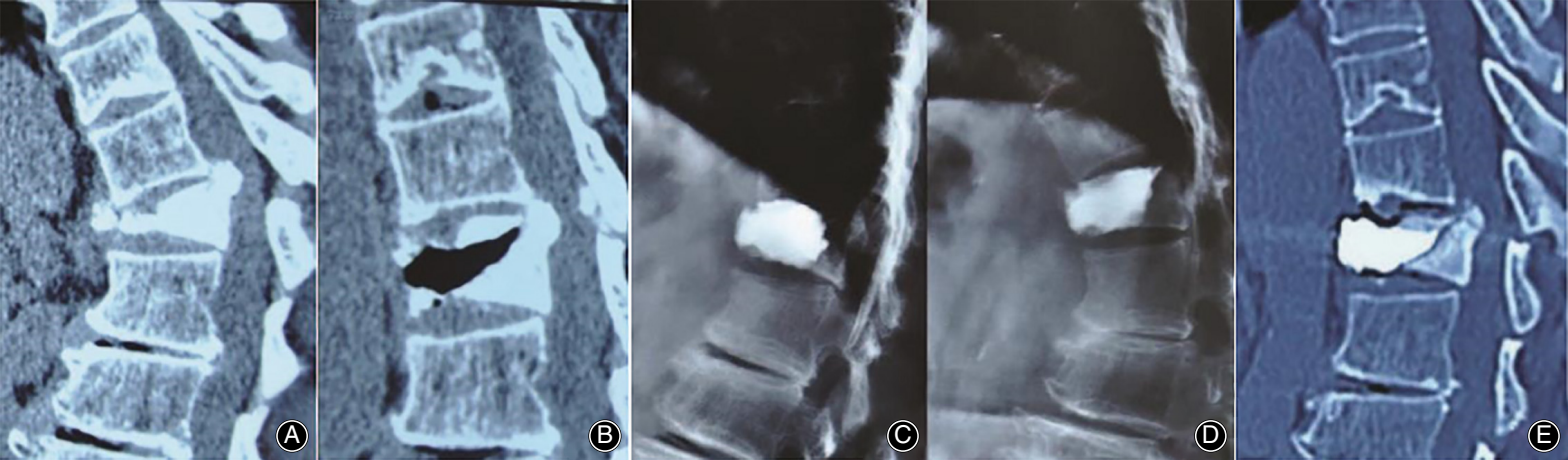
A typical patient with pattern II IVC from Zhang *et al*.^4^. (A) Preoperative hyperflexion lateral CT. (B) Preoperative hyperextension lateral CT. (C) and (D) Postoperative dynamic x‐ray shows the twelfth thoracic vertebra (T12) vertebra stability. (E) CT revealed the dislocation of cement one month after the PKP.

Pattern II, intravertebral vertical clefts traversed to anterior edge of the vertebral body affected. Most cases of this pattern are very dynamic and unstable, for the nonunion runs through the anterior column, even the anterior and middle columns, and lets the remaining rear structure of the vertebrae to possibly act as a hinge when the spine flexes forward and extends backward. Kyphosis in this pattern usually exacerbates when the patient stands up and alleviates when they lie flat. Cement may leak out of the vertebral body through the cleft, or may dislodge forward as a whole in the future. This process was well demonstrated in a case report by Zhang *et al*.^4^(Fig. 6).

Huang *et al*.^28^ found thermal osteonecrosis, foreign body reaction, and fibrotic wall formation in the cement–bone interface; the lack of revascularization and repair process within the vertebral bodies are the characteristic histopathologic findings of retrieved specimens from vertebroplasty with PMMA cement. These interface problems make cement merely a space occupying material without mechanical interlock and biocompatibility and, therefore, increase the potential for dislodgment or fragmentation. Anterolateral displacement of the bone cement and associated micro‐motion would result in breakdown of the anterior and middle column of the vertebral body, leading to further kyphotic deformity^20^.

The pattern of IVC is a vital factor, but not the only factor that should be considered in treating KD during the clinical practice. The following conditions should be taken into consideration when a KD patient is under therapy selection: neurological deficits requiring decompression, Kyphosis deformity requiring correction (Cobb angle greater than 30°), or dynamic instability (the difference of Cobb angle during dynamic radiography greater than 10°) requiring fixation operation. In this study, if the patient had any these conditions, they would be defined as type A; if intractable back pain was the only complaint without any of these conditions, they would be defined as type B. The general principle of treatment for KD was to enhance and support anterior column, and to fix and stabilize posterior column. The treatment strategies were as described above; in brief, LSF was performed in all type A patients, while SSF and PKP were optional in type B patients. Good results have been achieved in LSF and SSF groups. There were no serious intraoperative or postoperative complications, no loosening, displacement, or fracture of internal fixation at the final follow‐up, no neurologic deterioration was seen after the surgery. LSF and SSF are safe and effective for treating KD, and can achieve satisfactory correction of kyphosis and vertebral height, with pain relief and improvement in patient's daily life, with few complications.

But in PKP group, cement leakage into intervertebral space occurred in four patients; among them, three patients had recurrent lower back pain 3–9 months after the procedure. Although the VSA and ODI showed statistically significant differences between pre‐ and post‐operation and between pre‐ and final follow‐up (*P* < 0.05), we could observe that the VSA and ODI rebounded a little at the final follow‐up. This resulted in the differences between postoperative and final follow‐up values, which were also statistically significant (*P* < 0.05). We recommend that PKP should be chosen carefully if the IVC of the patient presents to be pattern I or II.

### *Limitations*

This study is limited in that the number of cases included was not enough, and more cases should be included in further research. Except for the patterns of IVC indicated in this study, other patterns of IVC might be discovered according to more cases. In addition, this is a retrospective study, and financial factors and preoperative medications may have influenced the result of this study.

### *Conclusion*

There are two main patterns of IVC related to the high risk of cement complications in minimally invasive treatments for KD. LSF and SSF are safe and effective for treating KD and can achieve the satisfactory correction of kyphosis and vertebral height, provide pain relief and improvement in patient's daily life, with few complications. PKP should be chosen carefully if the IVC of the patient presents to be pattern I or II. However, the performance of IVC in KD should be observed in more patients in the future study, and the long‐term clinical outcomes and radiological results require further evaluation.
